# The use of aged stool specimens for the detection of rotavirus

**DOI:** 10.4102/sajid.v35i1.113

**Published:** 2020-03-09

**Authors:** Karin de Bruyn, Elizabeth M.C. Theron, John B. Dewar, Richard M. Hendrick

**Affiliations:** 1 College of Agriculture and Environmental Science Laboratories, College of Agriculture and Environmental Science, University of South Africa, Roodepoort, South Africa; 2 Department of Life and Consumer Sciences, College of Agriculture and Environmental Science, University of South Africa, Roodepoort, South Africa; 3 Department of Agriculture and Environmental Sciences, College of Agriculture and Environmental Science, University of South Africa, Roodepoort, South Africa

**Keywords:** diarrhoea, enzyme immunoassays, rotavirus, specimen storage, specimen viability

## Abstract

**Background:**

Rotavirus is considered worldwide as one of the most important viral gastrointestinal infections, resulting in potentially life-threatening diarrhoea and death in children under the age of 5 years. Rotavirus can survive and remain infectious for long periods outside of the human body and can be easily transmitted via environmental surfaces.

**Method:**

Stool specimens that had been collected and stored since 2010/2011 at 2°C – 8°C instead of −20°C or −80°C were analysed to determine the viability of rotavirus in these specimens after 6 years of improper storage. The specimens were analysed using simple enzyme immunoassay (EIA) methods from two different suppliers at different times throughout the period (2012–2017).

**Results:**

The analysis showed similar detection results for the two EIA kits.

**Conclusion:**

The rotavirus can be detected after several years of incorrect storage with EIA kits.

## Introduction

In spite of the introduction of various rotavirus vaccine programmes, rotavirus infection remains one of the major causes of mortality in developing countries.^1^ It is considered a universal disease amongst young children regardless of hygiene, food and water quality.^2,3,4^ Rotavirus is spread through faecal–oral transmission, contaminated water and food, through direct contact with infected individuals or contaminated surfaces and possibly through respiratory secretions.^3^ The spread of this virus has also been shown to be associated with waterborne outbreaks because of its ability to survive in potable and recreational water sources.^5^

Rotavirus needs human cells to replicate. The virus is incredibly resilient in the environment and can remain infectious for weeks outside of the human body.^2^ In addition, rotaviruses are highly contagious, with only small amounts (10–100 particles) of virus needed to infect humans.^2^ According to Rzeżutka and Cook,^6^ transmission and spread of gastrointestinal viruses, such as rotavirus, is determined by how long they can survive outside of the host. If they are robust and can survive for long periods in the environment while still remaining infectious, their chances of spreading and causing disease are increased.

In a study conducted by D’Souza et al.,^7^ it was stated that low environmental temperatures between 4°C and 20°C are ideal for the survival of rotavirus, and that the virus can remain viable outside of the host for several months. The virus can remain stable and even infectious in environmental temperatures reaching 30°C for 2–5 months in storage. According to Moe and Shirley,^8^ rotavirus inactivation rates are low at 4°C, and the virus is able to survive at these temperatures. Rotaviruses have been shown to survive for longer on non-porous surfaces at 4°C and have remained infectious for up to 10 days.^6^ The virus infectivity is also persistent for up to 1 week under normal room temperatures.^8^

Rotavirus can also survive in freshwater at 4°C for up to 32 days and can survive in tap water for over 64 days.^6^ According to Espinosa et al.,^9^ rotavirus infectivity in groundwater samples has shown to be only slightly reduced after 60 days, and samples still showed slight infectivity for up to 7 months. The viral genome also remained stable in groundwater samples that were kept at 15°C. Rotavirus also remained infective after 64 days at 20°C in filtered raw water samples.^9^ Rotavirus ribonucleic acid could retain infectiousness because the virus capsid is resistant to protein degradation because of the three-layer protein capsid structure. In the study by Espinosa et al.,^9^ they concluded that rotavirus could remain infectious for several months in groundwater sources.

Rotavirus survival on lettuce, radishes and carrots ranges from 25 to 30 days when kept at a refrigerator temperature of 4°C and between 15 and 25 days when kept at a room temperature of 25°C.^10^ These results indicate that enteric viruses are very robust and well adapted to survive for long periods in different environmental conditions, thus underscoring the transmissibility of these pathogens. However, this also makes it possible to detect these viruses in specimens even if they have not been stored properly as might be the case in rural areas where access to cold storage facilities might be interrupted or difficult to maintain. More remote areas can thus benefit from these findings, as they do not always have access to optimum storage conditions but can still diagnose patient specimens. While specimens should be transported on ice, if they reach the laboratories at non-optimal temperatures, these specimens can still be analysed instead of being discarded or patients having to resubmit specimens.

The aim of this study was to determine if the specimens collected in 2010/2011 and stored at 2°C – 8°C are still viable for rotavirus detection/screening after years of storage at non-optimal temperatures, and if the virus can still be detected in the specimen by means of EIA kits. In addition, the re-testing of the same specimens with two different EIAs allowed a comparison of the accuracy and efficiency of the kits.

## Materials and methods

A total of 63 specimens, collected from children under the age of 5 who presented with diarrhoea symptoms, were received from a pathology company. Permission was received from the pathologist at the pathology practice to make use of collected stool specimens, and ethics clearance was issued by University of South Africa (UNISA). Data on collected stool specimens had no personal information about the patients, and each specimen was given a number to ensure confidentiality and anonymity of the participants in the study.

The ProSpecT™ Rotavirus Kit (Oxoid Ltd, Basingstoke, United Kingdom) and the DRG^®^ Rotavirus Ag Enzyme-linked immunosorbent assay (ELISA) kit (DRG Instruments GmbH, Marburg, Germany) were used to screen stool specimens for rotavirus as per the manufacturer’s instructions except that the specimens were diluted in water instead of the kit diluent. The reason for not using the diluent provided with the kits was that some of the specimens were already diluted in water for other testing. Thus, to ensure consistency of the testing protocols, all the remaining specimens were diluted with distilled water. The ProSpecT™ EIA plates were read on an ELx800 Absorbance Reader with Gen5 software (BioTek Instruments Inc, Winooski, VT, USA) at a 450 nm wavelength. The results were exported in a Microsoft Excel format. The DRG^®^ Rotavirus Ag ELISA plate was read at an optical density (OD) of 450/620 nm, with a Varioskan microplate reader and Flash SkanIt software (Thermo Fisher Scientific, Waltham, MA, USA) within 30 min. The cut-off values of each kit were determined as indicated by the manufacturer’s instructions.

The specimens were originally tested in 2010/2011 with rapid test strips, the Combi^®^ immunochromatographic test (ICT) strips (Coris BioConcepts, Gembloux, Belgium), by the pathology company and then again with the ProSpecT™ Rotavirus kit in 2012. A total of 63 samples were randomly selected, based on the amount of specimen available, and re-tested with a ProSpecT™ Rotavirus kit in 2014 and with a DRG^®^ Rotavirus Ag ELISA kit in 2017. Statistical validation of the results was achieved by making use of the chi-square test to determine if there were any associations between the different variables.

### Ethical considerations

Ethical approval was obtained from the Research Ethics Review Committee of the College of Agriculture and Environmental Sciences, University of South Africa (reference number: 2014/CAES/081).

## Results

A total of 63 specimens were tested, and the results are shown in Table 1. The specimens were originally tested with Combi^®^ ICT by a pathology company, and from these specimens positive and negative specimens were selected and re-tested with EIA kits from different manufacturers at different time periods. The comparison, as shown in Table 1, shows that for three of the specimens, the analysis done in 2017 gave positive results where it previously showed negative results.

**TABLE 1:** Comparison of enzyme immunoassay kits and rapid test strip results.

Observed values	Combi® ICT 2010/2011	ProSpecT™ 2012	ProSpecT™ 2014	DRG® 2017
Positive samples	50	50	50	53
Negative samples	13	13	13	10
**Total**	**63**	**63**	**63**	**63**

ICT, immunochromatographic test.

The calculated test statistics (χ^2^ = 0.684025) does not exceed the critical value (7.815), and thus we cannot reject the null hypothesis. This means that there is no association between the EIA kit used and the rotavirus results.

## Discussion

Ideally, faecal specimens containing rotavirus should be stored at −20°C for prolonged periods to maintain viability of viral particles for cultivation and genotyping analysis. However, in this study, rotavirus was detected in specimens even after 6 years of storage at 2°C – 8°C. Similar results were obtained in a study conducted by Fischer et al.,^11^ where specimens were unintentionally stored for two-and-a half months at temperatures above 30°C. The rotavirus strains remained stable and possibly still infectious in spite of improper storage. Rotaviruses have also been shown to remain stable for up to 32 months at 10°C,^11^ indicating that specimens can be kept and analysis performed in areas where electricity or power supply is a problem or access to cold storage is limited. This also highlights the fact that these specimens should be disposed of in a safe and proper manner so as not to cause unintentional exposure to the virus.

The rotavirus screening results were similar, except for three specimens (5%; 3/63) that were negative on Combi^®^ ICT and the ProSpecT™ but positive on the DRG^®^ kit. These results may indicate an increase in the sensitivity or an increase in the false positivity rate of the newer test. It should be noted, however, that the results could also be because of the fact that the specimens were diluted with distilled water and not the diluent supplied by the kits. This could account for the difference in sensitivities of these kits and should be considered a limitation of the study. However, it is important for manufacturers of diagnostic tests, such as rapid test strips and EIA, to regularly assess kit performance by undertaking additional quality control or eliciting customer feedback. This will ensure sustained sensitivity of their detection assays and increase the possibility of detecting the disease-causing organisms when present in lower concentrations.

The EIA tests evaluated in this study were all able to detect rotavirus in the stool specimens in spite of the poor storage conditions. In addition, although the EIA tests were from different manufacturers, they provided similar rotavirus results. The results not only show the efficiency of the EIA kits but also the prolonged stability of rotavirus in stool specimens. This prolonged stability ensures persistence in the environment and aids the spread of rotavirus through communities.

## Conclusion

Prolonged storage at improper temperatures does not affect the stability of rotavirus in stool specimens. The results showed that the virus could still be detected in stool specimens after being stored for 6 years at 2°C – 8°C instead of the recommended −20°C or −80°C. Possible recommendations include re-evaluation of shipping and storage conditions, as this could be done in a more cost-effective manner. Future research should include studies on more complex analysis techniques with these old samples so as to determine if results can still be obtained with methods such as polymerase chain reaction.
